# The survival outcomes of neoadjuvant sintilimab combined with chemotherapy in patients with locally advanced esophageal squamous cell carcinoma

**DOI:** 10.3389/fimmu.2022.1100750

**Published:** 2023-01-19

**Authors:** Huilai Lv, Chao Huang, Jiachen Li, Fan Zhang, Chunyue Gai, Zhao Liu, Shi Xu, Mingbo Wang, Zhenhua Li, Ziqiang Tian

**Affiliations:** Department of Thoracic Surgery, The Fourth Hospital of Hebei Medical University, Shijiazhuang, Hebei, China

**Keywords:** esophageal squamous cell carcinoma, pathological complete response, immune checkpoint inhibitors, sintilimab, survival outcomes

## Abstract

**Background:**

Neoadjuvant programmed death receptor-1 (PD-1) inhibitor combined with chemotherapy has been reported to improve the pathological response of locally advanced esophageal squamous cell carcinoma (ESCC), but the systematic report on survival follow-up is quite few. This study we will report the survival follow-up outcomes after a median follow-up of 21.1 months.

**Methods:**

This was a real-world retrospective study. Locally advanced ESCC patients treated with neoadjuvant sintilimab combined with albumin-bound paclitaxel and nedaplatin followed by surgery and completed at least 1-year follow-up were reviewed. The primary outcome was disease-free survival (DFS) at 24 months. The secondary outcome was overall survival (OS) at 24 months.

**Results:**

Ninety eligible patients were included in the analysis between July 2019 and October 2021. The median number of neoadjuvant cycles was 3 (range 2-4). All patients achieved R0 resection. With a median follow-up of 21.1 months (range 14.0-39.0), the median DFS and median OS had not reached, 2-year DFS rate was 78.3% (95%CI 68.8%-89.1%) and 2-years OS rate was 88.0% (95%CI 80.6%-96.0%). Postoperative pathological stage, pCR, MPR, tumor down-staging were significantly correlated with favorable survival outcome. Univariable and multivariable Cox regression analysis identified cycle number of neoadjuvant treatment as independent predictor of DFS.

**Conclusion:**

Our results preliminarily show a survival benefit of neoadjuvant sintilimab combined with chemotherapy in locally advanced ESCC.

## 1 Introduction

Esophageal cancer (EC) is one of the most common malignant tumors of digestive system in China (1, 2). According to the latest WHO epidemiological data, there were 324422 estimated new cases and 301135 estimated deaths of esophageal cancer in China in 2020, which were about half of the global cases (2). The predominant histological type of esophageal cancer in China is esophageal squamous cell carcinoma (ESCC), accounting for about 90% (3, 4). For locally advanced resectable ESCC, multidisciplinary comprehensive treatment strategies including neoadjuvant or perioperative treatment, and esophagectomy have been established.

Neoadjuvant chemotherapy and neoadjuvant chemoradiotherapy have been confirmed to significantly improve the survival of patients with esophageal cancer and was recommended by the National Comprehensive Cancer Network (NCCN) guidelines and the Chinese Society of Clinical Oncology (CSCO) guidelines (5–13). Programmed cell death-1 (PD-1) inhibitor combined with chemotherapy have shown promising outcomes and approved by regulatory in the first-line therapy of advanced esophageal and gastroesophageal junction (GEJ) carcinoma (14–19). Preliminary data from several small-sample studies showed that PD-1 inhibitor combined with chemotherapy have also great application prospect in the neoadjuvant setting, with the pathological complete response (pCR) rate 21.7% to 50% (20–32). The 2022 CSCO guideline recommend PD-1 inhibitor combined with chemotherapy (category 3) as one of the neoadjuvant treatment options (13). However, the systematic report on survival follow-up of neoadjuvant PD-1 inhibitor combined with chemotherapy is quite few. In previous real-world study, we reported the encouraging pathological response and good tolerability of neoadjuvant sintilimab combined with platinum and taxanes in resectable locally advanced ESCC: the pCR rate was 30.2%, the major pathological response (MPR) rate was 62.5%, the pathological downstaging rate was 74.0% (31). In this study, we analyzed the patients who were followed up for more than one year and report the survival outcomes after a median follow-up of 21.1 months.

## 2 Methods

### 2.1 Study design and patient selection

This study was designed to be a real-world retrospective study to investigate the survival outcomes of neoadjuvant sintilimab combined with chemotherapy in patients with locally advanced ESCC at The Fourth Hospital of Hebei Medical University. The study was approved by the Ethics Committee of our hospital and conducted in accordance with the 2013 edition of the Declaration of Helsinki. Written informed consent were waived. The inclusion criteria were included: patients with histologically confirmed locally advanced resectable ESCC, aged 18 years or older, both sexes, with clinical stage II-IVA, treating with neoadjuvant sintilimab combined with albumin-bound paclitaxel and nedaplatin followed by surgery, completed at least 1-year follow-up. Exclusion criteria included: having other anti-tumor treatments before or during the neoadjuvant treatment. Pretreatment baseline staging was according to American Joint Committee on Cancer (AJCC) 8th edition TNM staging system. Chest-abdominal contrast enhanced computed tomography (CT), esophageal enhanced magnetic resonance imaging (MRI), endoscopic ultrasound (EUS), and cervical ultrasound were performed. Position emission tomography positron emission tomography computed tomography (PET-CT) was also performed when necessary.

### 2.2 Treatment

All eligible patients completed 2-4 cycles of neoadjuvant treatment with sintilimab (200mg, I.V, D1), albumin-bound paclitaxel (260mg/m (2), I.V, D1) and nedaplatin (80mg/m (2), I.V, D1) of each 3-week cycle. Dose reductions were not permitted for sintilimab. Sintilimab was discontinued if a grade 2 or specific grade 3 treatment-related adverse event (TRAE) occurred during treatment and resumed when the TRAE reduce to grade 1 or below. Sintilimab was permanently discontinued if a specific grade 3 or grade 4 TRAE occurred. Chemotherapy was discontinued when patients experienced a grade 3–4 TRAE and resumed when the TRAE reduce to grade 1 or below. The chemotherapy dose at resuming was reduced to 75% of the initial dose when the grade 3–4 AE occurred for the first time and continued to reduce to 50% of the initial dose when the grade 3–4 AE reoccurred. The chemotherapy was permanently discontinued if the grade 3–4 AE reoccurred despite 2 dose reductions. Radiologic responses and clinical restaging were assessed every two cycles by the same means as the baseline. McKeown esophagectomy with two- or three-field lymphadenectomy was selected according to patients’ condition and performed for all suitable patients. Pathological examination was carried out according to the standard protocols. Pathological response was assessed by tumor regression grade (TRG) referred to the Becker system (33). The adjuvant regimens include albumin-bound paclitaxel and nedaplatin, sintilimab monotherapy, or sintilimab combined with albumin-bound paclitaxel and nedaplatin. The decision was made through the multidisciplinary board according to the original response to immune-chemotherapy and postoperative clinical conditions. The adjuvant regimen was decided by selected by multidisciplinary team according to the efficacy and safety of neoadjuvant treatment and postoperative recovery, including adjuvant albumin-bound paclitaxel and nedaplatin, sintilimab monotherapy, or sintilimab combined with albumin-bound paclitaxel and nedaplatin. pCR patients may not receive adjuvant treatment. Follow-up were routinely conducted every 3 months during first 2 years after surgery, and then every 6 months after 2 years.

### 2.3 Outcomes

The primary outcome was disease-free survival (DFS) at 24 months. DFS was defined as the time from the date of neoadjuvant treatment to recurrence or death by any cause. Disease recurrence included locoregional recurrence (LRR) and distant recurrence (DR) that occurred after R0 resection. Locoregional recurrence was defined as the recurrence within the esophagus, anastomosis or regional lymph nodes. Distant recurrence was defined as distant organ metastases, peritoneal carcinomatosis or recurrence within nonregional lymph node. The secondary outcomes were overall survival (OS) at 24 months. OS was defined as the time from the date of neoadjuvant treatment to death by any cause. Impact of pathological response (pCR, MPR and tumor downstaging) on DFS was exploratory analyzed. pCR (corresponds with Becker TRG 1a) was defined as no evidence of residual tumor cells in the resected primary tumor and axillary lymph nodes. MPR (corresponds with Becker TRG 1a+1b) was defined as 10% or fewer residual tumor cells in the primary tumor. Tumor downstaging was defined as a decrease in T or/and N of pre-surgery stage after neoadjuvant (ypTNM) or post-surgery pathological stage (pTNM) relative to baseline clinical stage (cTNM). Upstaging was defined as an increase in T or/and N of ypTNM or pTNM stage relative to baseline cTNM.

### 2.4 Statistical analysis

Statistical analysis was performed using SPSS software (IBM SPSS Statistics, RRID: SCR_016479 version 26.0) and R software (RRID: SCR_001905 version 4.0.0). The continuous variables were presented as median and range. The categorical variables were presented as number and percentage. The pCR, MPR, tumor downstaging and R0 resection rate with 95% CI were calculated using the Clopper–Pearson exact method based on binomial distribution. Median follow-up time was calculated using the reverse Kaplan–Meier method. DFS and OS and corresponding 95% CIs were estimated using the Kaplan-Meier method, and a log-rank test was used for comparisons between pathological response subgroups. Univariable and multivariable Cox regression models were used to estimate the effect of neoadjuvant treatment among subgroups according to baseline characteristics. All statistical testing is two-tailed and performed at the 5% significance level.

## 3 Results

### 3.1 Baseline characteristics

Ninety eligible patients were included in the analysis between July 2019 and October 2021. The major characteristics of the patients are shown in **Table 1**. Sixty-one (67.8%) were male, the median age was 65 years (range 49-78 years), most tumors were located in the middle esophagus (44.4%) and lower esophagus (42.2%), 48 (53.3%) patients had Eastern Cooperative Oncology Group (ECOG) performance status (PS) 0 and 42 (46.7%) had ECOG PS 1 or 2. Thirty-two (35.5%) patients had clinical stage II, 52 (57.8%) patients had clinical stage III, and 6 (6.7%) had clinical stage IVA.

**Table 1:** Patient baseline characteristics.

Characteristics	No. (%)
Age (years)	
Median (range)	65 (49-78)
<65	44 (48.9)
≥65	46 (51.1)
Sex	
Male	61(67.8)
Female	29(32.2)
Smoking	
Yes	37(41.1)
No	53(58.9)
Alcohol Drinking	
Yes	40(44.4)
No	50(55.6)
Tumor Location	
Upper Esophagus	12(13.3)
Middle Esophagus	40(44.4)
Lower Esophagus	38(42.2)
Clinical TNM Stage	
II	32(35.5)
III	52(57.8)
IVA	6(6.7)
Clinical T Stage	
3	87(96.7)
4a	3(3.3)
Clinical N Stage	
0	33(36.7)
1	41(45.6)
2	12(13.3)
3	4(4.4)
ECOG PS Score	
0	48(53.3)
1	34(37.8)
2	8(8.9)

### 3.2 Treatment outcomes

The median number of neoadjuvant cycles was 3 (range 2-4). Forty-two (46.7%) patients received 2 cycles of the neoadjuvant treatment, and another forty-eight (53.3%) received 3–4 cycles. Eighteen patients experienced chemotherapy dose reduction due to TRAEs. Five patients experienced treatment discontinuation due to TRAEs, including one patient because of Immune-mediated colitis, one patient because of liver Abnormalities, two patients because of hyperthyroidism, and two patients because of leukopenia and neutropenia. No treatment-related surgical delay or death was observed.

All 90 patients underwent scheduled surgery, all patients achieved R0 resection. The median interval between the end of neoadjuvant therapy and surgery was 34 days (range 21-95 days). The median number of resected lymph node was 29 (range 20-52). Seventy-five (83.3%) patients completed two field lymphadenectomy, and 15 (16.7%) completed three field lymphadenectomy. The median operation time was 303 minutes (range 142-563 minutes), and the median intraoperative blood loss was 150ml (range 100-2300 ml). The treatment responses are summarized in **Table 2**. Postoperative pathological analysis showed that the pCR (TRG1a) rate was 31.1% (95CI 21.8%-41.7%), MPR (TRG1a+1b) rate was 61.1% (95CI 50.3%-71.2%). The median length of hospital stay was 12 days (range 8-54 days).

**Table 2:** Radiologic and pathologic responses.

Variable	No. (%)
**R0 resection**	90(100.0)
TRG	
**TRG1a**	28(31.1)
**TRG1b**	27(30.0)
**TRG2**	16(17.8)
**TRG3**	19(21.1)
ypTNM stage	
**I**	47(52.2)
**II**	20(22.2)
**III**	15(16.7)
**IVA**	8(8.9)
ypT Stage	
**0**	33(36.7)
**1**	19(21.1)
**2**	12(13.3)
**3**	23(25.6)
**4a**	3(3.3)
ypN Stage	
**0**	51(56.7)
**1**	25(27.8)
**2**	8(8.9)
**3**	6(6.7)
**Downstaging (ypTNM VS. cTNM)**	71(78.9)
**Unchanged staging (ypTNM VS. cTNM)**	14(15.6)
**Upstaging (ypTNM VS. cTNM)**	5(5.6)
pTNM stage	
**0/I**	44(48.9)
**II**	19(21.1)
**III**	20(22.2)
**IVA**	7(7.8)
pT Stage	
**0**	33(36.7)
**1**	20(22.2)
**2**	12(13.3)
**3**	22(24.4)
**4a**	3(3.3)
pN Stage	
**0**	53(58.9)
**1**	22(24.4)
**2**	9(10.0)
**3**	6(6.7)
**Downstaging (pTNM VS. cTNM)**	70(77.8)
**Unchanged staging (pTNM VS. cTNM)**	13(14.4)
**Upstaging (pTNM VS. cTNM)**	7(7.8)

Sixty (66.7%) patients received adjuvant therapy, including 50 (55.6%) patients receiving sintilimab combined with chemotherapy, 5 (5.6%) patients receiving sintilimab, and 5 (5.6%) patients receiving chemotherapy. The median cycle of adjuvant sintilimab was 8 (range, 1-17), The median cycle of adjuvant chemotherapy was 2 (range, 1-4).

### 3.3 Survival outcomes

As of data cut-off, the median follow-up was 21.1 months (range 14.0-39.0). 15 (16.7%) patients had disease recurrence and 9 (10.0%) of them died, in addition, 1 (1.1%) patient died of non-cancer-related cause without recurrence. 2 (2.2%) patients experienced locoregional recurrence, 12 (13.3%) patients experienced distant recurrence, and 1 (1.1%) patient experienced concurrent locoregional recurrence and distant recurrence (**Table 3**). Of the 30 patients who were followed up for more than 24 months, only 3 recurred 15.8 months, 16.9 months and 20.8 months after treatment, respectively.

**Table 3:** Characteristics of patients with disease recurrence.

Patient NO.	Age	Sex	ECOG PS	Tumor Location	Cycle Numbers	cTNM (baseline)	pTNM	TRG	Metastatic Sites	DFS (months)	OS (months)
1	57	male	1	middle	2	T3N1M0	T2N1M0	TRG3	retroperitoneal lymph node	12.6	NR
2	51	male	1	middle	2	T3N2M0	T3N1M0	TRG2	liver	15.8	26.0
3	72	female	2	middle	2	T3N1M0	T3N2M0	TRG3	retroperitoneal lymph node	14.2	16.7
4	57	male	1	lower	2	T4aN2M0	T1N1M0	TRG1b	pretracheal lymph node, bone	11.6	18.7
5	66	male	1	lower	2	T3N0M0	T3N0M0	TRG3	Left hilar lymph node	10.2	14.3
6	69	male	2	lower	3	T3N3M0	T1N3M0	TRG1b	retroperitoneal lymph node	11.0	16.1
7	63	female	0	middle	3	T3N0M0	T0N0M0	TRG1a	Left supraclavicular lymph node, lung	16.9	NR
8	60	male	2	middle	2	T3N1M0	T3N1M0	TRG3	retroperitoneal lymph node, Left supraclavicular lymph node	20.8	NR
9	64	female	0	middle	3	T3N1M0	T3N1M0	TRG3	liver	20.8	NR
10	65	male	0	middle	2	T3N1M0	T0N1M0	TRG1b	bone	5.3	14.4
11	50	female	1	lower	2	T3N1M0	T3N3M0	TRG3	liver, lung	14.9	NR
12	71	female	0	middle	2	T3N2M0	T3N3M0	TRG3	liver	4.9	10.0
13	63	male	1	lower	3	T3N1M0	T2N1M0	TRG2	adrenal	12.1	NR
14	56	male	0	middle	2	T3N1M0	T2N1M0	TRG2	pretracheal lymph node	10.7	14.3
15	74	male	0	lower	2	T3N1M0	T4aN3M0	TRG3	lung	6.3	8.3

*NR, not reach.

**Figure 1:**
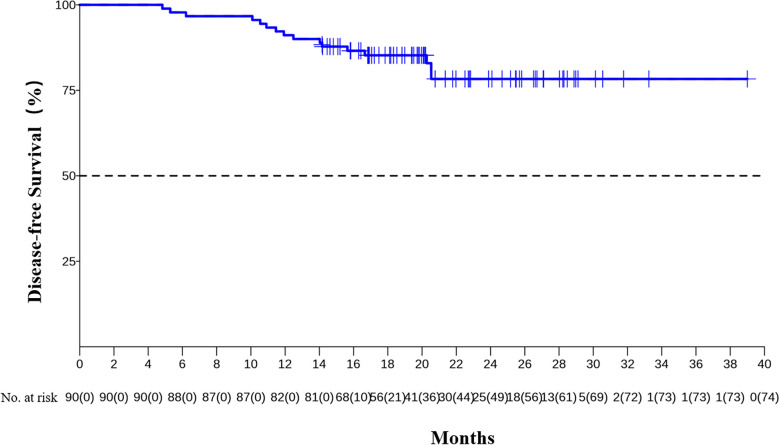
Kaplan-Meier estimates of DFS.

**Figure 2:**
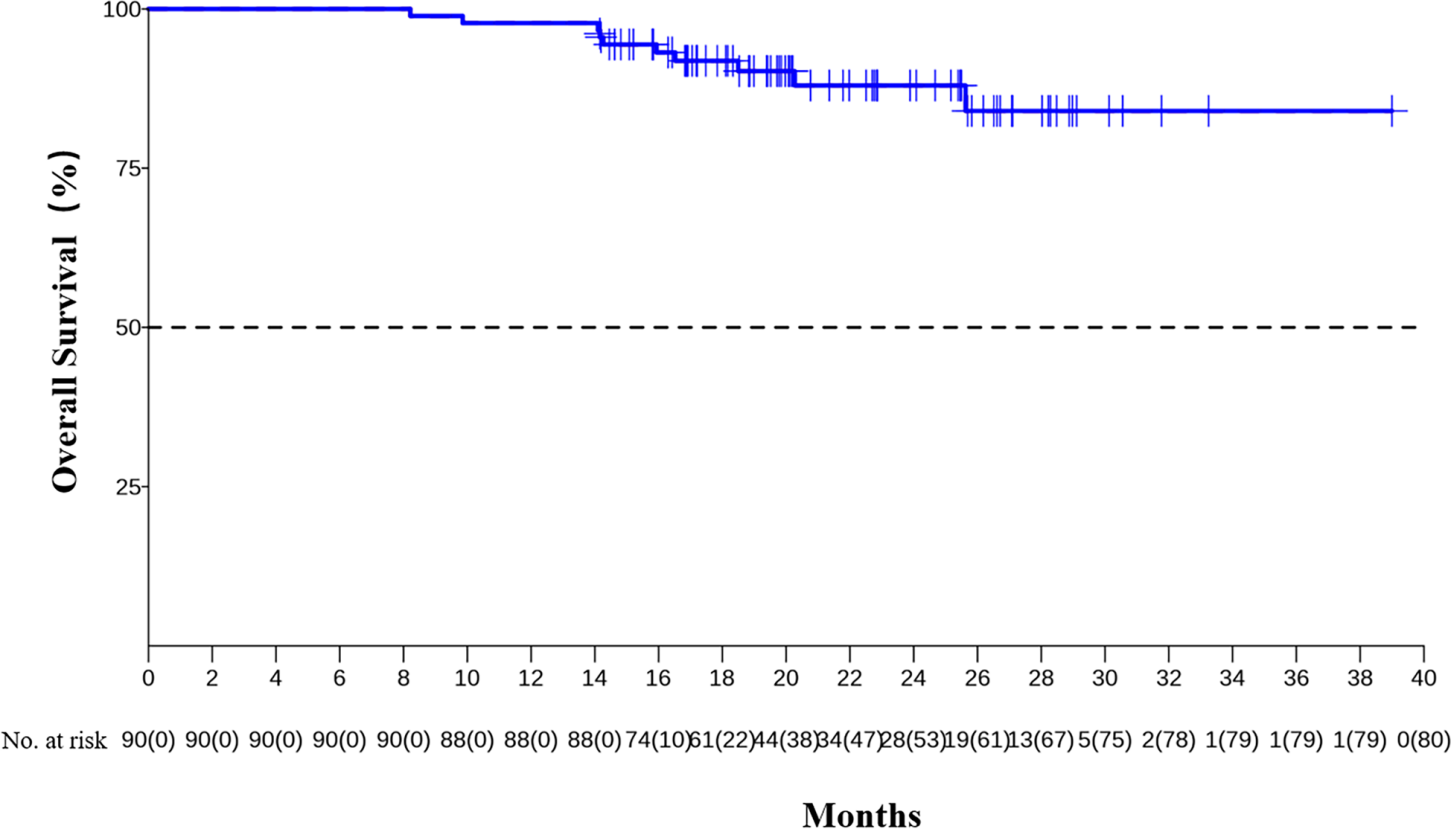
Kaplan-Meier estimates of OS.

**Figure 3:**
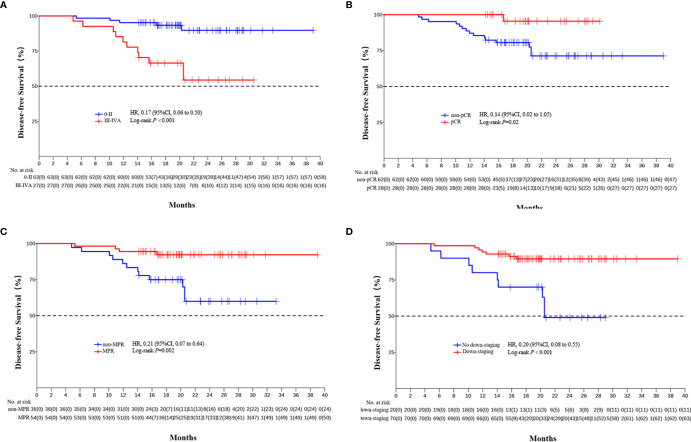
Kaplan-Meier estimates of DFS stratified by pathological responses. **(A)** DFS of the pathological stage 0-II group and the III/IVA group. **(B)** DFS of the pCR group and the non-PCR group. **(C)** DFS of the MPR group and the non-MPR group. **(D)** DFS of the tumor pathological down-staging group and not achieving tumor pathological down-staging group.

**Figure 4:**
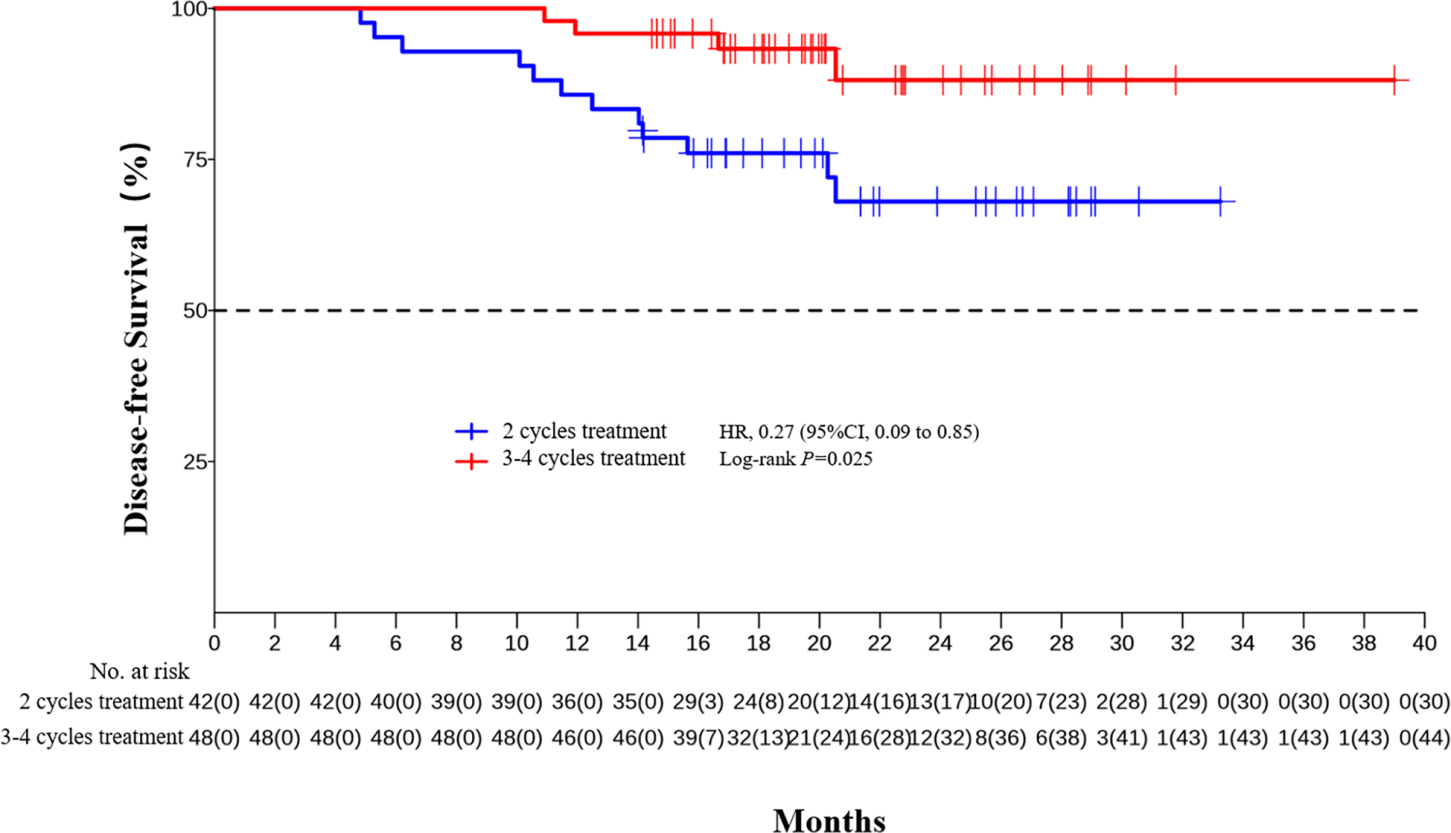
Kaplan-Meier estimates of DFS stratified by cycle number of treatment.

The median DFS and median OS had not been reached yet, 1-year DFS rate was 91.1% (95%CI 85.4%-97.2%), and 2-year DFS rate was 78.3% (95%CI 68.8%-89.1%). 1-years OS rate was 97.8% (95%CI 94.8%-100.0%), and 2-years OS rate was 88.0% (95%CI 80.6%-96.0%) (**Figures 1**, **2**). Subgroup analysis showed that postoperative pathological stage, pCR, MPR, tumor pathological down-staging were significantly correlated with survival outcome (**Table 4**, **Figure 3**). Univariable and multivariable Cox regression analysis according to baseline characteristics identified cycle number of neoadjuvant treatment as independent predictor of DFS (**Table 5**). Patients who completed 3–4 cycles of neoadjuvant treatment increase survival compared to those received 2 cycles, 2-year DFS rate was 88.1% (95%CI 76.9%-100%) and 68.0% (95%CI 53.1%-85.6%) respectively (**Figure 4**).

**Table 4:** Comparisons between pathological response subgroups.

	Events, No./total No.	2-year DFS (95% CI)	HR (95% CI)	P value
Pathological stage 0-II	5/63 (7.9%)	89.7% (95%CI 81.0%-99.4%)		
Pathological stage III/IVA	11/27 (40.7%)	54.4% (95%CI 36.9%-80.1%)	0.17 (0.06-0.50)	<;0.001
pCR	1/28 (3.6%)	95.5% (95%CI 87.1%-100.0%)		
Non-pCR	15/62 (24.2%)	71.3% (95%CI 59.2%-85.8%)	0.14 (0.02-1.05)	0.02
MPR	4/54 (7.4%)	92.2% (95%CI 85.1%-99.9%)		
Non-MPR	12/36 (33.3%)	59.9% (95%CI 43.7%-82.2%)	0.21 (0.07-0.64)	0.002
Tumor pathological down-staging	7/70 (10.0%)	89.5% (95%CI 82.4%-97.2%)		
Not achieving down-staging	9/20 (45.0%)	49.0% (95%CI 29.8%-80.5%)	0.20 (0.08-0.55)	<;0.001

**Table 5:** Univariable and multivariable Cox regression analysis according to baseline characteristics.

Variable	Events, No./total No.	Univariable analysis		Multivariable analysis*	
		HR (95% CI)	P value	HR (95% CI)	P value
Age (years)					
<65	10/44 (22.7%)				
≥65	6/46 (13.0%)	0.53 (0.19-1.46)	0.221		
Sex					
Male	10/61 (16.4%)				
Female	6/29 (20.7%)	1.26 (0.46-3.48)	0.650		
Tumor Location					
Lower Esophagus	7/38 (18.4%)				
Middle Esophagus	9/40 (22.5%)	1.09 (0.41-2.95)	0.862		
Upper Esophagus	0/12 (0)	–	0.977		
Clinical T Stage					
3	15/87 (17.2%)				
4a	1/3 (33.3%)	1.83 (0.24-13.94)	0.559		
Clinical N Stage					
0	3/33 (9.1%)				
1	9/41 (22.0%)	2.59 (0.70-9.56)	0.153	2.25 (0.59-8.56)	0.236
2-3	4/16 (25.0%)	3.01 (0.67-13.45)	0.150	2.55 (0.57-11.71)	0.228
ECOG PS Score					
0	6/48 (12.5%)				
1	7/34 (20.6%)	1.67 (0.56-4.96)	0.360	1.37 (0.45-4.17)	0.577
2	3/8 (37.5%)	2.91 (0.73-11.65)	0.131	2.29 (0.55-9.54)	0.256
Cycle Numbers					
2	12/42 (28.6%)				
3-4	4/48 (8.3%)	0.27 (0.09-0.85)	0.025	0.29 (0.09-0.92)	0.035

*Multivariable analysis included the following baseline characteristics: clinical N stage, ECG PS score and cycle numbers of neoadjuvant treatment.

## 4 Discussion

These survival Outcomes, after a median follow-up of 21.1 months, preliminarily show a survival benefit of neoadjuvant sintilimab combined with chemotherapy in locally advanced ESCC. In recent years, more and more studies have confirmed the encouraging pathological response and manageable safety of neoadjuvant PD-1 inhibitor combined with chemotherapy (20–32). Now, it is of concern whether the encouraging pathological response can be translated into survival benefit. This is a relatively large sample and systematic report on survival follow-up.

As to the survival follow-up outcomes of neoadjuvant PD-1 inhibitors and chemotherapy in the ESCC, there are also four other small sample studies. A single-arm, prospective trial of 23 enrolled patients showed after a median follow-up of 13.77 months (IQR: 9.7–17.6), 5 (25%) of the 20 patients who received surgery experienced disease recurrence or metastasis ranging from 4 to 12 months after surgery (25). Another single-arm, prospective trial of 47 enrolled patients showed after a median follow-up of 14.6 months (IQR, 11.3- 24.0 months), the 1-year OS was 90.8%, and the 1-year DFS was 68.3%, and patients who achieved MPR had improved DFS (*p*=0.050, HR=0.35) and OS (*p*=0.066, HR=0.16) (32). A retrospective study of 34 enrolled patients showed the DFS rate was 86.4% at 12 months, 70.4% at 24 months, and the OS rate was 92.8% at 12 months, 83.2% at 24 months after a median follow-up of 14.8 months (range 3.8–26.5) (29). Another retrospective study of 47 enrolled patients showed that the 1-, 2-year DFS were 95.7%, 80.7%, the 1-, 2-year OS rates were 95.7%, 83.2% (27). In our study, more patients were included and the followed-up time was nearly two years. The results showed that 1-year DFS rate of neoadjuvant sintilimab combined with chemotherapy was 91.1%, 2-year DFS rate was 78.3%, 1-years OS rate was 97.8%, 2-years OS rate was 88.0% with a median follow-up of 21.1 months. Overall, all these follow-up outcomes preliminarily show survival benefit and further confirm the prospect of neoadjuvant PD-1 inhibitor combined with chemotherapy.

Some studies reported that combined radiotherapy based on chemotherapy can mainly improve local pathological response, but may be poorly effective at controlling occult systemic metastasis (11, 34–36). The Chemoradiotherapy for Oesophageal Cancer Followed by Surgery Study (CROSS) showed the reduction in distant progression in the neoadjuvant chemoradiotherapy plus surgery group was significant than the surgery alone group during the first 24 months of follow-up but not thereafter (36). The NEOCRTEC5010 clinical trial also showed the reduction in distant progression was not significant after the first 24 months (11). The LRR, DR, and overall recurrence rates of neoadjuvant sintilimab combined with chemotherapy during the first 24 months decreased numerically compared with neoadjuvant chemoradiotherapy (11, 36, 37). Moreover, the presence of the whole tumor during neoadjuvant PD-1 inhibitor combined with chemotherapy allows a triggering of a broader T cell response due to a larger repertoire of tumor antigen exposure, then establish systemic immune surveillance and destruction of micrometastases (38, 39). So the better postoperative pathological response from neoadjuvant PD-1 inhibitor combined with chemotherapy may translate into a long-term survival benefit. This has been confirmed in the neoadjuvant immunotherapy of lung cancer (40–43).

In the present study, subgroup analysis showed that postoperative pathological stage, pCR, MPR, tumor down-staging were significantly correlated with survival outcome. The results preliminarily indicate that pCR and MPR can be used as alternative indicators or predictor of survival for neoadjuvant PD-1 inhibitor combined with chemotherapy, which is consistent with previous findings in neoadjuvant chemotherapy and neoadjuvant chemoradiotherapy (44–48), and we will further confirm it through long-term follow-up.

The optimal number of neoadjuvant treatment cycles remains to be established for ESCC. For neoadjuvant chemotherapy, a randomized phase II trial showed three courses of DCF led to a relatively higher rate of pCR (15.3% vs. 9.1%, P = 0.212) compared to the two-course (49). Another randomized phase II trial showed two- and three-course of DCF have the comparable histological responses (P=0.898), and the 2-year PFS rate was also comparable between the two groups (71.4 vs. 71.1%, P = 0.669) (50). Our previous studies have shown that the pCR rate were significantly higher in patient completed 3-4 cycles of neoadjuvant therapy than those completed 2 cycles, (47.9%, 95% CI, 33.3%–62.8% vs. 12.5%, 95% CI, 4.7%–25.2%, p = 0.0003). In the present study, multivariable Cox regression analysis also identified cycle number of neoadjuvant treatment as independent predictor of DFS. Patients who completed 3–4 cycles of neoadjuvant treatment increase survival compared to those received 2 cycles (2-year DFS rate 88.1% vs. 68.0%). Long term follow-up and randomized study are still needed to further confirm the benefits of extending the treatment cycle of neoadjuvant PD-1 inhibitor combined with chemotherapy to 3-4.

There are several limitations to this study. First, this is a retrospective study which may cause biases. Second, more samples are needed to further confirm the conclusion. Third, our study only reported a short-term survival outcome, longer follow-up is necessary to evaluate the long-term clinical benefits of neoadjuvant PD-1 inhibitor combined with chemotherapy for locally advanced ESCC.

## Data availability statement

The original contributions presented in the study are included in the article/supplementary material. Further inquiries can be directed to the corresponding author.

## Ethics statement

The studies involving human participants were reviewed and approved by The Ethics Committee of The Fourth Hospital of Hebei Medical. Written informed consent for participation was not required for this study in accordance with the national legislation and the institutional requirements.

## Author contributions

ZT and HL designed the study. CH and SX collected the data. HL and CH analyzed and interpreted the data. CH, JL, FZ, CG, ZL, MW, and ZHL carried out the clinical treatment and management of the patients. ZT and HL prepared the final draft. All authors contributed to the article and approved the submitted version.
